# Perception and Representation of Lexical Tones in Native Mandarin-Learning Infants and Toddlers

**DOI:** 10.3389/fpsyg.2017.01117

**Published:** 2017-07-21

**Authors:** Rushen Shi, Jun Gao, André Achim, Aijun Li

**Affiliations:** ^1^Département de Psychologie, Université du Québec à Montréal Montréal, QC, Canada; ^2^Phonetics and Speech Science Lab, Institute of Linguistics, Chinese Academy of Social Sciences Beijing, China

**Keywords:** lexical tones, infant speech processing, lexical representation, phonological neutralization, language acquisition

## Abstract

We investigated the perceptual development of lexical tones in native tone-learning infants during the first 2 years of life, focusing on two important stages of phonological acquisition: the preverbal and vocabulary explosion stages. Experiment 1 examined monolingual Mandarin-Chinese-learning 4- to 13-month-olds' discrimination of similar lexical tones in Mandarin, Tone 2 (T2, rising) vs. Tone 3 (T3, low-dipping). Infants were habituated to exemplars of one tone (either T2 or T3), and tested with new exemplars of the habituated tone vs. the contrasting tone. Results show that looking time increased for the contrasting tone, but not for new exemplars of the habituated tone, suggesting that infants discriminated the two tones as separate categories. Furthermore, infants' discrimination of the tones was comparable across ages. Experiment 2 tested whether tones are distinguished in toddlers' lexicon. Monolingual Mandarin-learning 19- to 26-month-olds were presented with pairs of objects while one was named. Targets were familiar words bearing T2 or T3, either correctly pronounced (CP) or mispronounced (MP) in tone. We found that word recognition was equally successful in CP and in MP trials when T2 was mispronounced as T3 and T3 as T2, indicating that T2 and T3 are confusable. In contrast, recognition failed when T2 and T3 words were mispronounced as Tone 4 (T4, falling), showing that T4 was represented as a distinct category. Results show that toddlers have difficulty encoding similar tones distinctly in known words. The T2-T3 contrast is particularly challenging because of Tone 3 Sandhi, which changes T3 to T2 when it precedes another T3. At the stage when toddlers track the meaning of T2 and T3 words and track the sandhi alternations, they seem to overgeneralize the two tones as variants of one functional category, reflecting perceptual organization at the level of phonemic learning.

## Introduction

Within the first year of life infants make significant advances in acquiring the native-language sound system. They initially perceive both native and non-native consonant and vowel contrasts, and gradually reorganize their perception according to the native language categories (e.g., Werker and Tees, 1984; Kuhl et al., 1992; Polka and Werker, 1994). In particular, during the second half of the first year of life, infants' sensitivity to non-native contrasts declines, while native contrasts continue to be discriminable. This reorganization is largely driven by distributional analysis of the input (e.g., Maye et al., 2002; Anderson et al., 2003).

To establish the full phonological system of the native language, infants would subsequently need to understand the relevance of the phonetic categories for distinguishing word meaning, and to acquire a lexicon as well as the associated phonemic structure. During early word learning shortly after the first year of life, infants confuse similar-sounding segments in certain tasks. For example, in Stager and Werker (1997) 14-month-old infants confused /b/ and /d/ in a word-object association task. The confusion seems to be due to task difficulty and the processing demand of word learning, since infants at this age succeeded in perceiving phonetic details in studies using more sensitive word-learning tasks (Ballem and Plunkett, 2005; Yoshida et al., 2009). Further, similar-sounding segments in familiar words are distinguished from an early age. Several studies have shown that even at the stage when the receptive lexicon is small, recognition is affected if a consonant or a vowel of a familiar word is mispronounced (Swingley and Aslin, 2002; Fennell and Werker, 2003; Mani and Plunkett, 2007). For instance, infants' looking time was affected when *ball* is mispronounced as *doll* (Fennell and Werker, 2003); when *car* is mispronounced as *cur*, visual fixation to the named object picture decreases (Swingley and Aslin, 2002). Moreover, toddlers showed graded sub-segmental representations for familiar words in a sensitive mispronunciation task (White and Morgan, 2008), similar to adults. Taken together, native segmental categories seem well distinguished in the early lexicon, especially for words that infants know well, although phonetically similar segments may be confusing for infants in certain word learning tasks.

Lexical tones are phonemic and are found in many languages (e.g., in Asia). Much less research has been conducted on early perceptual development of lexical tones. The present study investigated the perception and representation of native lexical tones in Mandarin-Chinese-learning children at two important stages of learning: the preverbal stage, and the vocabulary explosion stage. Specifically, we inquired (1) whether native tone-language-learning preverbal infants, who know a limited number of words and have not yet acquired a sophisticated phonological system, discriminate lexical tone contrasts, and if they do, (2) whether toddlers subsequently represent the tonal contrasts in familiar words. These questions thus concern the development from early phonetically based tonal discrimination to later representation of tonal contrasts in the lexicon. The latter is essential for acquiring a mature phonological system.

**Figure 1:**
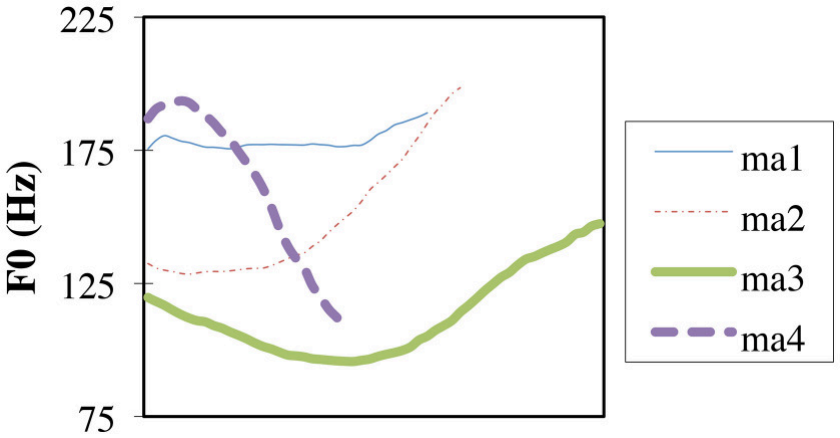
F0 trajectories of the four Mandarin lexical tones (high: Tone 1 *ma1* “mother,” rising: Tone 2 *ma2* “hemp,” low-dipping: Tone 3 *ma3* “horse,” falling: Tone 4 *ma4* “to curse”) in citation, produced by a male speaker. In the 5-level pitch notation by Chao (1930) the four tones are 55, 35, 214, and 51.

Mandarin-Chinese has four lexical tones: high (T1), rising (T2), low-dipping (T3), and falling (T4). In Chao's 5-level pitch notation (Chao, 1930) the four tones are 55, 35, 214, and 51. The fundamental frequency (F0) is the primary acoustic correlate of lexical tones. The tone-bearing unit is the syllable (Xu and Wang, 2001). Other acoustic cues to tonal contrasts also exist. For instance, as shown in Figure 1, T1 and T4 are shorter than T2 and T3, with T3 being the longest in isolation (e.g., Xu, 1997). T3 is often produced with a distinct creaky voice at low pitch. Among all the tonal contrasts in Mandarin, the T2-T3 contrast is widely considered to be the most similar in pitch pattern. Nevertheless, the contrast is supported by multiple acoustic cues. Even non-tone-speaking teenagers can discriminate this contrast based purely on acoustic processing (Pierce et al., 2014).

The tones in Mandarin differ in their phonological structure, with T3 being the most complicated. T3 is subject to sandhi (the Tone 3 Sandhi rule), according to which T3 is realized as a T2-like rising tone (35 in Chao's notation, i.e., it is neutralized to T2) when T3 immediately precedes another T3, and T3 is a low tone (11 in Chao's notation) before any other tone. Utterance-final and citation T3 (see Figure 1) has the most complex contour (214 in Chao's notation). In other words, the rising, the low, and the complex contour are the three variants of T3. Tone 3 Sandhi is a rule that applies generally across lexical items that bear T3 as the underlying tonal representation. Sandhi alternations also occur with other tones, although they only apply to a few specific lexical items. For example, the negation particle *bu4* and the numeral *yi1* (“one”), both highly frequent, go through sandhi alternations depending on context: they are realized as T2 when preceding T4, and as T4 when preceding all other tones. These item-specific alternations need to be learned as exceptions to the general non-alternating pattern of T4 and T1 words, unlike the learning of the Tone 3 Sandhi rule. T2, T4, and the utterance-final and citation variants of T3 are contour tones, whereas T1 and the low variant of T3 are level tones. Across tone languages, contour tones are considered more complex than level tones (Yip, 2002). For instance, a rising contour tone can be described in terms of combined tone height features (e.g., LH for T2 in Mandarin, with L and H representing the Low and High features) whereas a level tone can be represented with a single tone height feature (e.g., H for T1 in Mandarin). The feature representations for T3 are complex, with the utterance-final and citation variant as LLH (or L plus a post-lexical floating H, depending on theories), and non-final variants as L and LH. The T2-T3 contrast is hence the most phonologically complex one in Mandarin.

In the 3 sections below we first review previous research on preverbal tone-learning infants' discrimination of native and non-native lexical tones. Next, we discuss studies on infants' and toddlers' tonal processing in word segmentation, word learning and word comprehension tasks. Finally, we present the hypotheses of our present study on Mandarin learners' discrimination of two native tones at the preverbal stage and their perception of the two tones in familiar words at the vocabulary explosion stage.

### Tone-learning infants' discrimination of lexical tones during the first year of life

Compared to consonants and vowels, much less is known about infants' discrimination of tones during the initial stages of learning, and only a few studies have tested preverbal tone-learning infants' perception of native tones (Harrison, 2000; Tsao, 2008; Yeung et al., 2013). Harrison (2000) was the first to test the discrimination of lexical tones in preverbal babies. Using the Conditioned Headturn Procedure, he showed that 6- to 8-month-old Yoruba-learning infants discriminated synthetic tones similar to the high tone vs. the mid tone in Yoruba, and their performance was consistent with that of adult native listeners.

Yeung et al. (2013) familiarized 4- and 9-month-old infants with one Cantonese tone (either high-rising or mid-level, i.e., 25 and 33 in Chao's notation), and the two tones were presented in three types of test trials: the familiarized tone, the contrasting tone, and both (alternating). Cantonese-learning infants did not show any differential looking times to the three types of test trials after being familiarized to the 33-tone. After they were familiarized with the 25-tone, they showed different looking times for alternating trials vs. 25-tone trials, although looking in 33-tone trials did not differ from looking in either alternating trials or 25-tone trials. Infants thus showed partial evidence supporting the discrimination of the tonal contrast. These results are difficult to interpret, as the patterns were not consistent across conditions and trials. The authors' predicted preference for alternating over non-alternating trials was not systematically observed. In this kind of task, discrimination is interpreted indirectly from preference. Infants may discriminate the contrast and prefer the more dynamic alternating trials; or they may discriminate the contrast but prefer the more familiar non-alternating trials. A systematic group preference for one type of test trials (for example, alternating over non-alternating) would be clear support for successful discrimination. However, lacking a systematic preference, as is the case in one of the familiarization groups in Yeung et al. (2013), does not necessarily mean a lack of discrimination.

In addition to Cantonese-learning infants Yeung et al. (2013) also tested Mandarin-learning 4- and 9-month-olds' perception of those two Cantonese tones (25-tone and 33-tone), which are similar to Mandarin T2 (rising) and T3 (low-dipping). After being familiarized to the 33-tone, Mandarin-learning babies showed no looking difference in the test phase, similar to the response of Cantonese-learning babies. After the 25-tone familiarization, looking was longer in 25-tone trials than in 33-tone trials, and longer in alternating trials than in 33-tone trials, but no looking difference was observed between 25-tone and alternating trials. Their preferential pattern differed from that of Cantonese-learning infants. Similar to the Cantonese babies, Mandarin-learning babies showed evidence of discrimination only in one of the familiarization conditions, with a complex pattern of preference. As discussed earlier, the non-predictability of their results was likely due to the nature of their task, which tested preference, but not necessarily discrimination. We suggest that the habituation task might be better suited to directly reveal discrimination. In such tasks infants are habituated to one member of a contrast, and then tested with the same habituated member and the contrasting member. Because habituation reflects a decrease in interest over time, a looking recovery to the new member, but not to the habituated member, is predicted when infants can discriminate the contrast. Conversely, if they cannot discriminate the contrast, they should show no looking increase upon hearing the new member relative to the old member during the test phase. In the present study we tested Mandarin-learning babies' discrimination of native tones using a habituation task.

Like the Cantonese contrast in Yeung et al. (2013), the Thai rising vs. low contrast is also similar to the T2-T3 contrast in Mandarin. Using the Conditioned Headturn Procedure, Mattock and Burnham (2006) showed that 6- and 9-month-old Chinese infants discriminated this Thai contrast, indicating that they might have assimilated the Thai contrast to their native contrasts (25-tone vs. 33-tone in Cantonese, or T2 vs. T3 in Mandarin).

Only one previous study has tested infants' discrimination of native tones in Mandarin. Using the Conditioned Headturn Procedure, Tsao (2008) tested Taiwan-Mandarin-learning 10–12-month-olds' discrimination of the T1-T3, T2-T3, and T2-T4 contrasts. Infants discriminated T1-T3 (73% correct) better than T2-T3 (61%) and T2-T4 (58%), and the performance of T2-T3 and T2-T4 were comparable. The superior T1-T3 discrimination was expected. Even non-Mandarin adults find these two tones perceptually distinct (So and Best, 2010). Their F0 height and trajectories are non-overlapping. T2 and T3 are generally considered more similar, with the F0 onset being relatively low for both. In citation, T2 and T3 both move up in F0 toward the offset. T2 and T4 are acoustically more dissimilar than T2-T3, as they involve opposite F0 trajectories (see Figure 1). However, the T2-T4 and T2-T3 contrasts were discriminated equivalently in Tsao's study, and both were less discriminable than T1-T3. In their task each infant was first taught to respond to a tonal change in a contrast, and the stimuli used for the teaching then served as the stimuli for testing that infant. Only infants who passed the training criterion were included in the test phase. Their experiment was designed for testing the relative discriminability of the three contrasts after the training. It would be interesting to test whether the tonal contrasts can be discriminated spontaneously, i.e., entirely based on infants' prior experience with the native language. In the present study we directly tested whether Mandarin-learning preverbal infants can discriminate native tones without any training.

### Lexical tones in toddlers' developing lexicon

Around the age of 1 year, children start building a lexicon and develop a sophisticated phonological system associated with the lexicon. In addition to encoding consonant and vowel contrasts, tone-language children need to encode the lexical tone of words. A recent study suggests that infants close to 1 year of age distinguish native lexical tones when recognizing words. Specifically, in an auditory speech segmentation/recognition task Singh and Foong (2012) first familiarized English-Mandarin bilinguals with isolated word forms, and then tested the infants with passages containing the target forms. They found that at 11 months of age infants recognized the Mandarin target forms in passages only when the forms matched the familiarized forms in tone, but not when the tone was mismatched, similar to the results of monolingual Mandarin-learning infants in Shi (2009); however, when familiarized and tested with English stimuli, the bilingual infants at this age ignored lexical-tone-like pitch changes in target words and recognized the words regardless of whether their pitch matched or mismatched with the familiarized form in tone. In a subsequent study 18- and 24-month-old Mandarin-English bilinguals encoded T2 (rising) and T4 (falling) distinctly when learning to map novel objects to novel words (Singh et al., 2014). This tonal contrast was also distinguished during word learning by monolingual English-learning infants at 14 months of age in Hay et al. (2015) and at 18 months in Singh et al. (2014), but not at 17–19 months in Hay et al. (2015). Nevertheless, although the 17-to-19-month-old English learners in Hay et al. (2015) failed to encode the T2-T4 distinction during word learning, they were still able to discriminate the contrast in an auditory habituation task, suggesting that sensitivity to tonal contrasts remains more acute for acoustic-phonetic based discrimination than for phonemic based lexical encoding. In a similar auditory habituation study (Shi et al., 2017) the discrimination of T1 (high) and T4 (falling) showed no decline in French-learning infants from 4 to 11 months of age.

How are lexical tones represented in toddlers' familiar words? A few studies have addressed this question, primarily with children older than 2.5 years of age, who have acquired a reasonable-sized lexicon. In Singh et al. (2015) Mandarin-English bilinguals aged 2.5–3.5 years distinguished the Mandarin T1-T2, T1-T4, and T2-T4 contrasts during familiar word comprehension. They looked less at the named object when its tone was mispronounced than when it was correctly pronounced. The same effect was shown for T1-T4 in Mandarin-speaking preschoolers; however, these children failed to detect the mispronunciations between T2 and T3 (Singh et al., 2017).

Wong et al. (2005) examined tonal recognition in monolingual Mandarin-speaking children, using a picture-pointing task. Three-year-olds were presented with familiar words, including tonal minimal pairs. Recognition accuracy was high for T1, T2, and T4 targets (nearly 90%), lower for T3 targets (69%). The errors were mostly mis-perception of T3 as T2. Interestingly, the confusion was unidirectional; T2 was rarely mis-perceived. This asymmetry seems to be related to Tone 3 Sandhi, which neutralizes T3 to T2. The T2-T3 asymmetry was also observed in adult Mandarin listeners in a recent ERP study (Li and Chen, 2015), in which mismatch negativity effects were greater and earlier when the stimuli presentation changed from T2 to T3 than when the change was from T3 to T2. That is, the presentation of T3 in the latter case automatically activated T2 as a variant of T3, causing a weak response when T2 was subsequently heard. The authors noted that this weak response was comparable to within-category tone processing.

The T3 targets in Wong et al. (2005) were utterance-final, where the tone sandhi should not happen. Children's confusion of T3 as T2 thus suggests a partial understanding of Tone 3 Sandhi, i.e., an over-neutralization of T3 to T2 without understanding the appropriate context. Phonological neutralization often occurs between similar segments. For example, the word-medial /t/ and /d/ in *latter* and *ladder* in American English are neutralized as a flap. Syllable-final obstruents become devoiced in German (e.g., /d/ neutralized to /t/). Similar segments such as /t/ and /d/ share many phonetic features and acoustic properties. In general, dissimilar segments (e.g., /b/-/h/) are less likely to be subject to neutralization. Tone 3 Sandhi is likely related to the fact that T3 and T2 are acoustically similar. The differentiation of the two tones at the lexical level might therefore be challenging for children due to Tone 3 Sandhi.

### The present study

Considering the scarcity of data on the acquisition of native lexical tones during the initial 2 years of life, the present study examined Mandarin-learning infants' and young toddlers' perception of T2 and T3 in Mandarin. These two tones are interesting because they are acoustically similar and may be affected by the Tone 3 Sandhi rule. We thus focused on two stages of learning. In Experiment 1 we tested whether preverbal babies, who are either prior to or at the beginning of building a lexicon, can discriminate T2 and T3 in a habituation/dishabituation task. At this stage, tone learning should be largely based on the distributional properties of the acoustic patterns of tonal categories in the native language, or on other mechanisms independent of an infant knowing a lexicon (e.g., Yeung and Werker, 2009; Feldman et al., 2013). We hypothesized that at this stage infants' organization of the tones should be simpler, and they should be able to perceive tones based on pure auditory-phonetic processing. Following this stage, children face a harder task: they must build a sophisticated phonemic system, which requires them to encode tonal (in addition to segmental) distinctions across words in their lexicon. Do toddlers represent the phonetically similar and neutralization-prone T2 and T3 distinctly for known words? The status of lexical tones for words familiar to young toddlers below age two has not been studied previously in online comprehension tasks. Thus, in Experiment 2 we used this task to test whether toddlers, who begin to have a reasonable-sized lexicon, distinguish the phonetically similar, neutralization-prone T2-T3 contrast as well as the dissimilar, non-neutralizable T2-T4 and T3-T4 contrasts for familiar words. We note that the T2-T4 and T2-T3 contrasts are equally discriminated by Mandarin-learning infants (Tsao, 2008) at 10–12 months of age. In the present study we hypothesized that the additional factor of lexical neutralization due to Tone 3 Sandhi might lead to the confusion of T2 and T3 for familiar words in young toddlers.

## Experiment 1

### Methods

#### Participants

Participants were 20 Mandarin-learning 4- to 13-month-olds residing in Beijing (mean: 08;29 days; range: 4;22–13;20; girls: 13). The infants were monolingual Beijing-Mandarin (i.e., standard Mandarin) learners. Seven other infants were tested but were excluded from the analysis due to fussiness (4) and experimenter errors (3). Our interest here was to inquire generally whether Mandarin-learning infants at the preverbal stage can discriminate T2 and T3. We therefore treated our infants as one single group. We decided to set the youngest age at 4 months, since in previous research tone-learning infants from 4 months of age showed evidence of discriminating lexical tones (Yeung et al., 2013). Moreover, tonal discrimination in previous studies did not change across age during the first year of life for tone-learners (Mattock and Burnham, 2006; Yeung et al., 2013).

#### Stimuli

We chose the syllable *can* /ts^h^an/ in T2 (*can2* “disabled”) and T3 (*can3* “tragic”) because the words are unknown to preverbal and early verbal infants (and absent in the Mandarin early vocabulary corpus of Hao et al., 2008). The decision to use unknown words was important, as our goal in this experiment was to assess infants' early discrimination ability without any possible influence of familiar words. A female native speaker of Mandarin recorded many repetitions of the words in a lively voice. Overall, *can3* tokens were longer than *can2* tokens. We carefully selected a subset of *can2* and *can3* tokens which overlapped in duration. The final stimuli were 13 tokens of *can2* and 13 tokens of *can3*. T2 tokens were on average 718 ms (range: 631-806; SD: 63), and T3 tokens 717 ms (range: 630–802; SD: 63). Moreover, the tokens were adjusted to have comparable amplitude. Thus, T2 and T3 here were more similar acoustically than usual. These controls enabled us to better assess the contribution of F0 to infants' discrimination of T2 and T3. Our initial plan was to conduct a further experiment including additional acoustic cues if infants could not discriminate the tones in Experiment 1. Table 1 shows the F0 measures of the stimuli. The values of the measures indicate that for T2, F0 increased greatly and consistently from the onset region to the offset of the contour, whereas the F0 contour remained relatively low for T3, with a center dip. The pattern is similar to the examples in Figure 1. The maximum F0 occurred at the tonal offset for both tones. The time point of the minimum F0 (i.e., inflection point) along the contours differed with respect to tones. Specifically, the minimum F0 for T2 occurred around the tonal onset (on average 7.85% from the beginning of the tone), followed by a continuous increase. On the other hand, the F0 of T3 decreased from the beginning to a minimum value toward the middle part of the tone (on average 43.12% from the onset). The dip in F0 was accompanied mostly by a distinct creaky voice. In sum, T2 and T3 tokens differed highly significantly in nearly all of the F0 measures, as shown in Table 1.

**Table 1:** Acoustic measures (means and standard deviations) of the T2 (rising) and T3 (low-dipping) stimuli in Experiment 1.

	**Tone 2 (Rising)**	**Tone 3 (Low-dipping)**	**Independent *t*-tests (2-tailed)**
Average F0 (Hz)	288.35 (30.38)	203.69 (16.93)	*t*_(24)_ = 8.776; *p* = 0.000
F0 at tone onset (Hz)	240.72 (29.99)	222.01 (14.78)	*t*_(24)_ = 2.017; *p* = 0.055
F0 at tone offset (Hz)	398.95 (36.58)	258.14 (32.74)	*t*_(24)_ = 10.341; *p* = 0.000
Minimum F0 (Hz)	235.24 (28.34)	130.20 (25.9)	*t*_(24)_ = 9.865; *p* = 0.000
Maximum F0 (Hz)	401.20 (39.97)	258.51 (32.99)	*t*_(24)_ = 9.927; *p* = 0.000
Time point of minimum F0 (%)	7.85 (7.73)	43.12 (8.68)	*t*_(24)_ = −11.002; *p* = 0.000

The visual stimulus for all trials was a colorful checkerboard-like image. The attention-getter was a jumping star along with bird singing sound.

#### Procedure

Infants were tested individually in a sound-attenuated chamber. The child sat on the parent's lap, facing a central monitor that displayed the visual stimuli. Loudspeakers adjacent to both sides of the monitor simultaneously played auditory stimuli. A computer in the neighboring room controlled the presentation of the audio-visual stimuli and recorded the child's looking times. A researcher blind to the stimuli and design observed the infant and started each trial when the child looked at the monitor. Parents heard masking music from noise-cancelation headphones.

#### Design

Each habituation and test trial was started when the infant looked at the front central monitor, and terminated when she looked away for at least 2 s or when the maximum trial length (about 21 s) elapsed. Between trials, the attention-getter was automatically presented to attract the infant back to the monitor. Each infant was habituated to seven tokens of one tone, either *can2* or *can3*. The seven tokens of one tone were presented randomly without replacement, and the set was repeated (with tokens always in a random order) until the infant became habituated. The six other tokens of each tone were reserved for test trials. The inter-stimulus interval (ISI) within each trial was 1,000 ms. When the total looking time of three consecutive habituation trials declined to 50% of the first three habituation trials, the habituation criterion was reached, and the test phase began. All infants heard the same test stimuli, in two types: Same (new tokens of the habituated tone) and Different (the non-habituated tone). The order of the trial types was counterbalanced across infants. The use of new exemplars for the Same tone in the test phase was important for our design: if infants increased their looking time upon hearing the exemplars of Different tone (relative to their looking during the last habituation trial), but not upon hearing new exemplars of the Same tone (relative to their looking during the last habituation trial), the response would indicate category discrimination. On the other hand, if infants increased looking equally in both the Same and Different test trials (relative to the last habituation trial), this response would simply indicate the detection of any new tokens rather than the discrimination of tonal categories.

### Results and discussion

We calculated the looking times (in seconds) of the test trials and the last habituation trial. Because the data of two of these three measures were significantly skewed (beyond two standard errors) across babies, transformation was needed before the analysis of variance (Csibra et al., 2016). To bring the skewness below one standard error within each trial type, we log-transformed (base 10) the data after subtracting a constant (1.3) from each looking time. This transformation corrected the skewness and made the data acceptably symmetrical for all three measures. A 2 × 3 ANOVA was then conducted, with the Habituation Tone (T2 vs. T3) as the between-subject factor and Trial Comparison (Last Hab, Same, Different) as the within-subject factor. The results showed a significant effect of Trial Comparison, *F*_(2, 36)_ = 3.952, *p* = 0.028, but no effect of Habituation Tone, *F*_(1, 18)_ = 0.014, *p* = 0.907, and no interaction of these factors, *F*_(2, 36)_ = 0.851, *p* = 0.435. That is, infants who were habituated to either T2 or T3 responded in the same fashion.

**Figure 2:**
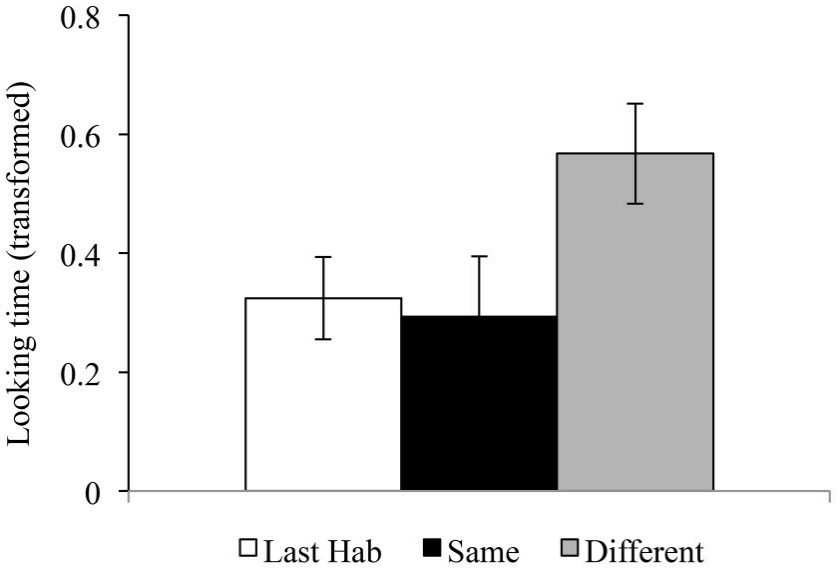
Experiment 1. Looking times (means and standard errors) of the Last Habituation trial, and the Same and Different test trials. Back transformation to looking times (in seconds) gives 3.41, 3.26, 4.99, respectively, for the three means in the figure.

Given the significant effect of Trial Comparison in the above analysis, the trial types were then analyzed in paired *t*-tests. The results revealed longer looking for Different (mean = 0.567, *SD* = 0.374, *SE* = 0.084) than Last Hab (mean = 0.324, *SD* = 0.311, *SE* = 0.069) [*t*_(19)_ = 2.465, *p* = 0.023], and for Different than Same (mean = 0.293, *SD* = 0.458, *SE* = 0.102) [*t*_(19)_ = 2.676, *p* = 0.015], but no difference for Same and Last Hab [*t*_(19)_ = −0.359, *p* = 0.724], all 2-tailed and uncorrected, as shown in Figure 2. Moreover, none of these pairwise differences correlated with age in days (|*r*| ≤ 0.262, *p* ≥ 0.265), suggesting that infants across ages (4–13 months) responded similarly.

Since both the Different and Same trials presented new exemplars after habituation, the results support category discrimination. Infants only increased their looking time upon hearing a new tonal category, but not upon hearing new exemplars of the habituated tone.

The results of Experiment 1 show that the phonetically similar T2-T3 contrast is discriminable at the pure phonetic level by preverbal Mandarin-learning babies. Our next question was whether this similar contrast is subsequently distinguished in words at a stage when children have established a sizable lexicon.

## Experiment 2

In Experiment 2 we chose to test toddlers aged 19–26 months, an age range characterized by vocabulary explosion. Toddlers of this age should have a reasonable-sized lexicon and are engaged in the learning of more advanced phonological knowledge. We presented toddlers with T2 and T3 familiar words. Besides correct pronunciations (CP), two types of tonal mispronunciations (MP) were presented: acoustically similar MPs (T2 mispronounced as T3, i.e., T2-to-T3; T3 mispronounced as T3, i.e., T3-to-T2) and dissimilar MPs (T2 mispronounced as T4, i.e., T2-to-T4; T3 mispronounced as T3, i.e., T3-to-T4). The similar MPs were relevant for neutralization (related to Tone 3 Sandhi) whereas the dissimilar MPs were not. We tested whether the two types of MPs were equally perceivable during word comprehension.

We used both the similar (T2 vs. T3) and dissimilar contrasts (T3 vs. T4, T2 vs. T4) to reveal how T2 and T3 are represented in the developing lexicon. We needed to include the dissimilar contrasts because they would likely show a mispronunciation effect, thus allowing us to confirm that a possible lack of a mispronunciation effect for the similar T2-to-T3 and T3-to-T2 changes would not be because of any peculiarity of the task. We hypothesized that although the T2-T3 contrast was easily discriminable during early infancy at the acoustic-phonetic level, toddlers might not represent this contrast distinctly for words due to the complexity of the tonal system at the lexical level and the sandhi rule related to the two tones. Furthermore, we hypothesized that T2 and T3 should be represented distinctly from T4, since T4 is acoustically dissimilar from either tone and there is no sandhi rule affecting the T2-T4 and T3-T4 contrasts.

### Methods

#### Participants

Participants were 64 monolingual Beijing-Mandarin-learning 19- to 26-month-olds residing in Beijing (mean: 21;29; range: 19;01–26;26; girls: 26). The data of 31 other toddlers were excluded due to fussiness (16), no interest in the task (9), parental interference (6), and researcher error (1). Children at this age should have acquired a sizable vocabulary according to the report of Hao et al. (2008). In their corpus the mean expressive vocabulary size of Beijing-Mandarin-learning children was 168 words (*SD* = 114) words at 19 months of age, and 376 (*SD* = 189) at 26 months of age.

#### Stimuli

Stimuli included monosyllabic T2 and T3 words (*yang2* “sheep,” *wan3* “bowl”) for the key trials. These key words are familiar to toddlers, as they appear in the majority of Mandarin-learning toddlers' production by 19 months of age in the early vocabulary corpus of Hao et al. (2008). Hao et al. (2008) did not collect data on toddlers' receptive vocabulary. Nevertheless, they reported both receptive and productive vocabularies for younger infants, with the former greatly exceeding the latter. For example, they reported that 16-month-old infants' mean productive vocabulary was 17 words, whereas their mean receptive vocabulary was 116 words. We can therefore infer that most toddlers in the Hao et al. database must be able to comprehend our key words by 19 months of age.

We also created two types of mispronunciations for these key words: 1) similar: T2 were mispronounced as T3 (i.e., the word *yang2* (“sheep”) was mispronounced as *yang3*: MP-*yang3*), and T3 as T2 (i.e., the word *wan3* (“bowl”) was mispronounced as *wan2*: MP-*wan2*); 2) dissimilar: T2 as T4 (i.e., *yang2* mispronounced as *yang4*: MP_*yang4*) and T3 as T4 (i.e., *wan3* mispronounced as *wan4*: MP_*wan4*). We note that the MP forms are existing words in Mandarin, but they are mostly unfamiliar to young children. In particular, the words *yang3* (“oxygen”), *wan2* (“pill”), *yang4* (“appearance”) and *wan4* (“wrist/ankle”) are uncommon object labels for toddlers and are all absent in the early vocabulary corpus of Hao et al. (2008).

In addition, we included 16 other familiar words as fillers^1^ (e.g., *hua1* “flower,” etc.) to make the task interesting to toddlers [see details in Appendix A (Supplementary Material)].

The same female Mandarin-Chinese speaker as in Experiment 1 recorded the speech stimuli in a sound-attenuated chamber. The final stimuli included two tokens for each target word and one token of the instruction utterances *kan!* (“look!”) and *zai nar?* (“Where is it?”).

For the key words, the CP mean duration was 614.25 ms (*SD* = 177.25), and the MP 518.75 ms (*SD* = 127.13). Tone 2 tokens were on average 459 ms (*SD* = 6.98) in length, Tone 3 tokens 743.75 ms (*SD* = 29.98), and T4 tokens 447.75 ms (*SD* = 39.61). Appendix B (Supplementary Material) shows the F0 trajectories of the first token of T2 and T3 words.

Visual stimuli were colorful pictures of objects for key words, filler words, and distractors. A picture of a laughing baby accompanied by the sound of a baby's laughter served as the attention-getter between trials.

#### Procedure and design

The equipment and room setup were the same as in Experiment 1. Infants were tested individually in the same sound-attenuated chamber as in Experiment 1. We used a within-subject design. Each test trial presented the images of two objects simultaneously on the far left and far right side of a 42-inch monitor; during a trial one object was named (i.e., the target), and the other unnamed (i.e., the distractor). The key trials presented the key words as the target in four CP trials (two CP-*yang2* trials; two CP-*wan3* trials), two similar MP trials (MP_23: one MP-*yang3* trial, one MP-*wan2* trial), and two dissimilar MP trials (MP_4: one MP-*yang4* trial, one MP-*wan4* trials), for a total of eight trials. MP_23 referred to trials in which T3 was mispronounced as T2, and trials in which T2 was mispronounced as T3. These trials tested whether the similar contrast of T2 vs. T3 were confusable to children in both directions. MP_4 referred to trials that presented T2-to-T4 or T3-to-T4 mispronunciations, which tested whether T2 and T3 were perceived as distinct from T4.

**Figure 3:**
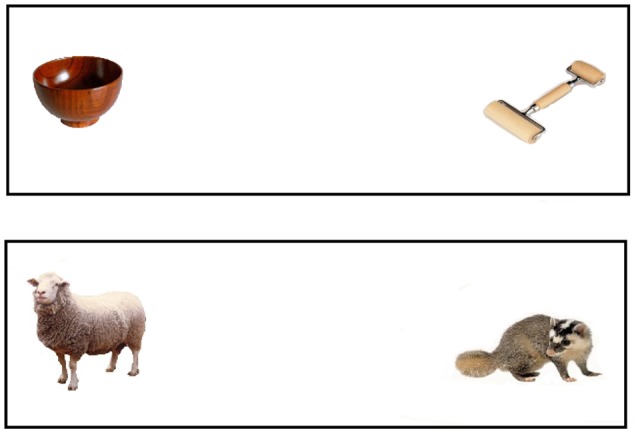
Images for the objects of key test trials in Experiment 2.

Images of two unfamiliar objects for which children have no words, a roller (painting tool) and a badger, were distractors in key trials in which they were paired, respectively, with the target images (bowl and sheep) (see Figure 3). To control for animacy, the roller was paired with the bowl, and the badger with the sheep. We used the unfamiliar distractors to make the measure more sensitive, as this would more likely lead children to decrease looking to the target upon hearing similar-sounding mispronunciations (White and Morgan, 2008).

The remaining were filler trials, in which the targets were always correctly pronounced. Trial order was quasi-randomized with the constraints that adjacent trials did not contain the same objects, and that no more than three consecutive trials presented targets with the same tone or on the same side. Key trials were always separated by filler trials. Four quasi-randomized orders were created. Toddlers were assigned randomly to four groups, and each group was tested with one of the four orders [see Appendix B (Supplementary Material)].

All trials were constructed with the same timeline. Images of two objects appeared for 2.1 s in silence, followed by the utterance *kan!* (“look!,” 458 ms) and then a 442 ms silence. The target word began exactly after 3 s from the trial onset, and *zai nar?* (“Where is it?”) began 1 s later, followed by the second presentation of the target word starting at the end of 5 s. The object pictures stayed for the whole trial of 6.5 s.

### Results

Videos of participants were coded offline by another researcher blind to the stimuli and design of the experiment using an in-house computer program. The coding was done at 25 frames/sec. For each frame, the looking was coded as left, right or elsewhere. We analyzed the 360–2,000 ms window from the onset of the first presentation of the target word, as in previous studies (e.g., Swingley and Aslin, 2002). The starting point of 360 ms was to account for the time needed for the child to initiate an eye movement. Within this window, the proportion of looking to target (PLT) was calculated by dividing the total looking time to the target by the sum of the looking times to the target and to the distractor.

A one-way repeated measure ANOVA was conducted, with Pronunciation (CP vs. MP_23 vs. MP_4) as the within-subject factor. The results revealed a significant effect of Pronunciation [CP: mean = 0.61, *SD* = 0.17, *SE* = 0.02; MP_23: mean = 0.64, *SD* = 0.28, *SE* = 0.04; MP_4: mean = 0.48, *SD* = 0.27, *SE* = 0.03; *F*_(1.819, 114.581)_ = 7.773, *p* = 0.001, Greenhouse-Geisser corrected].

**Figure 4:**
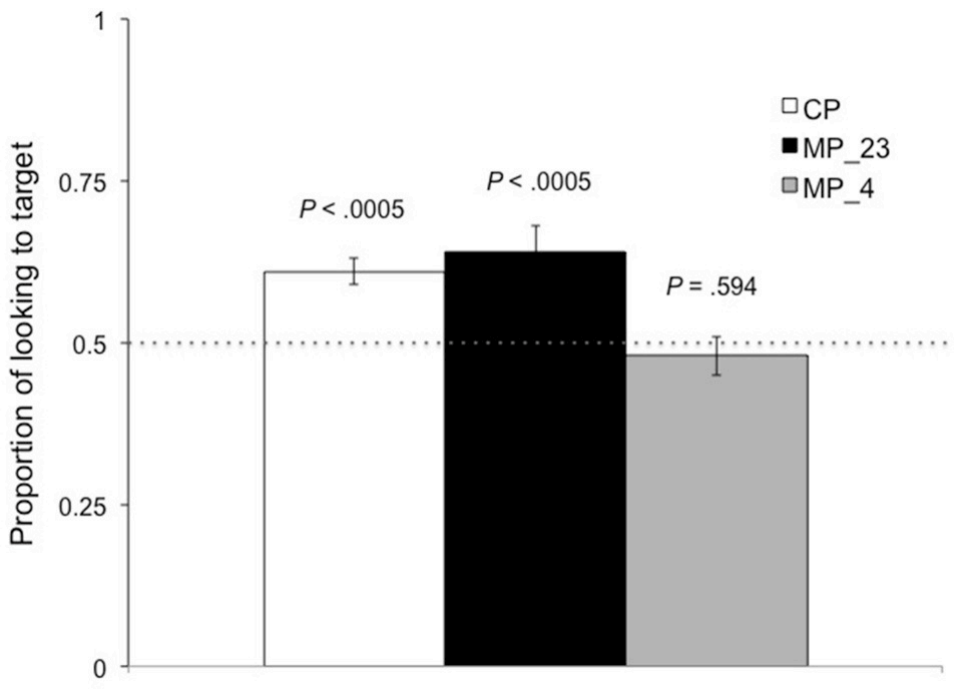
Experiment 2. PLTs (proportion of looking to target) during the analysis window of the CP trials (correct pronunciation) and MP trials (MP_23: T2 mispronounced as T3, T3 mispronounced as T2; MP_4: both T2 & T3 mispronounced as T4).

Subsequent pairwise comparisons were conducted using two-tailed *t*-tests. PLTs (proportion of looking to target) in CP and MP_23 did not differ from each other (*p* = 0.722), but both were higher than MP_4 (*p* = 0.001, *p* = 0.004). PLTs in both CP trials and MP_23 trials were significantly above the 0.5 chance level [*t*_(63)_ = 5.131, *p* < 0.0005; *t*_(63)_ = 3.898, *p* < 0.0005], whereas the PLT (proportion of looking to target) in MP_4 trials were at chance [*t*_(63)_ = −0.535, *p* = 0.594]. The results are shown in Figure 4. Given that the age range of our toddlers was from 19 to 26 months, we further explored whether toddlers' tonal perception during word comprehension changed within this age range. In particular, we analyzed the correlation between age and each pairwise comparison (i.e., age with “CP minus MP_23”; age with “CP minus MP_4”; age with “MP_23 minus MP_4”). The results showed no significant correlation between age and the pairwise comparisons (*r* = 0.225, *p* = 0.077; *r* = −0.051, *p* = 0.691; *r* = −0.131, *p* = 0.302), suggesting that toddlers across ages in our sample responded similarly to the test trials.

**Figure 5:**
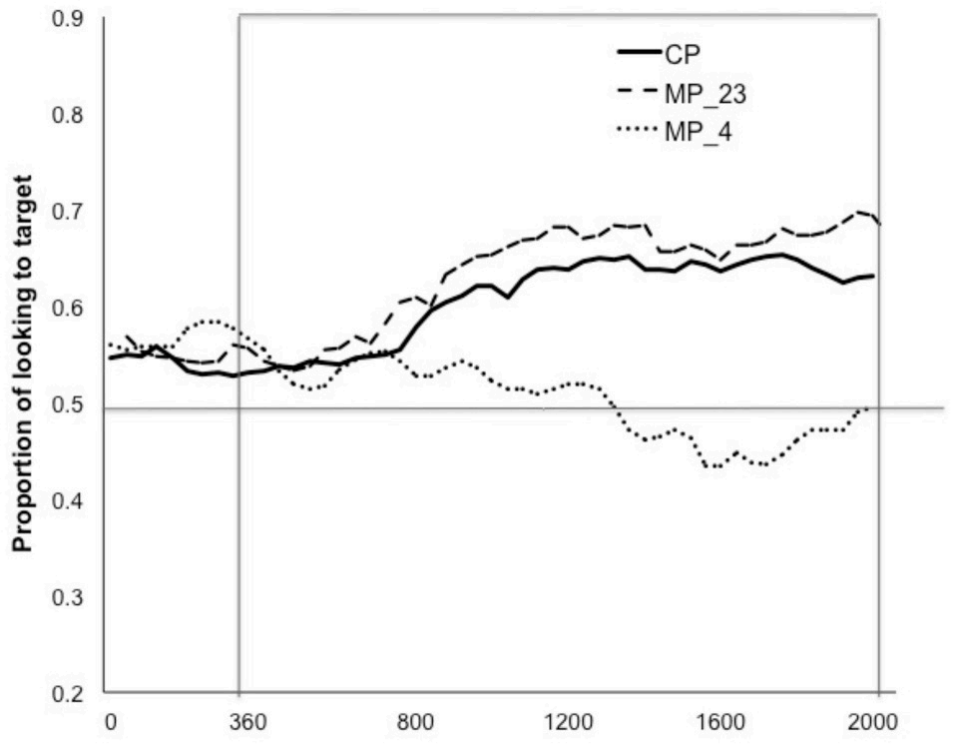
Experiment 2. The looking timecourse during the analysis window of the three test trials. The horizontal axis shows the onset of the target word at 0 and the analysis window from 360 ms to 2,000 ms.

Thus, children recognized the targets equally well in both CP and similar MP trials, but not in dissimilar MP trials. Figure 5 shows the looking timecourse during the analysis window, revealing that the recognition patterns in CP and similar MP trials were comparable, both diverging from the recognition pattern in the dissimilar MP trials. PLTs (proportion of looking to target) in the three trial types before naming, that is, in the window just preceding the target word onset (the same size as the post-onset analysis window) within the same trial, were comparable (CP: mean = 0.50, *SE* = 0.02; MP_23: mean = 0.54, *SE* = 0.03; MP_4: mean = 0.49, *SE* = 0.03) (*p* > 0.4) and were not different from chance (*p* ≥ 0.23), 2-tailed.

We further analyzed the specific tones of the key words in a 2 × 3 ANOVA, with Pronunciation (CP, MP_23, MP_4) and Tone (T2 vs. T3 targets) as within-subject factors. Since Tone 3 Sandhi involves a unidirectional T3-to-T2 change, a significant Pronunciation x Tone interaction was expected if the unidirectionality affected children's responses. In that case, we could then predict that children should detect the T2-to-T3 mispronunciation, but not the T3-to-T2 mispronunciation. No such interaction was expected if children had an overgeneralized representation (i.e., treating T2 and T3 as one functional category). Results showed again a significant effect of Pronunciation [*F*_(1.746, 80.339)_ = 4.251, *p* = 0.022, Greenhouse-Geisser corrected], but no significant main effect of Tone [*F*_(1, 46)_ = 2.960, *p* = 0.092], and crucially, no Pronunciation x Tone interaction [*F*_(1.864, 85.742)_ = 0.006, *p* = 0.992, Greenhouse-Geisser corrected]. The lack of any interaction with Tone indicates that responses to T2 and T3 targets followed the same patterns (i.e., confusion of T2 vs. T3 and T3 vs. T2; discrimination of T2 vs. T4 and T3 vs. T4). Recognition of the targets in CP trials (PLTs: T2 mean = 0.57, T2 *SD* = 0.25; T3 mean = 0.65, T3 *SD* = 0.198) and both T2-to-T3 MP trials (PLT: mean = 0.59, *SD* = 0.34) and T3-to-T2 MP trials (PLT mean = 0.67, *SD* = 0.36) was equally successful.

## Discussion

Lexical tones are an important part of the phonological system in many languages. The goal of the present study was to understand the acquisition of native lexical tones during the initial stages of development. We focused on children at two important stages of phonological acquisition during the first 2 years of life: preverbal babies, who have a limited vocabulary, and toddlers, who have a reasonable-sized lexicon. Experiment 1 demonstrates that Mandarin-learning preverbal babies can discriminate acoustically similar tones in their native language—namely, T2 vs. T3, which exhibit similar pitch trajectories. Notably, babies discriminated the two tones even though we eliminated the duration and amplitude cues. These results suggest that Mandarin-learning infants during the first year of life are highly sensitive to the pitch patterns of the two tones.

During the second year of life infants engage in more active word learning, and their lexicon grows significantly, particularly when they reach the vocabulary explosion stage several months before age two. In Experiment 2 we asked whether the similar T2-T3 contrast, which was perceivable at the preverbal stage, was subsequently encoded in toddlers' lexicon. Our results show that toddlers did not detect mispronunciations of T2 as T3, and T3 as T2; proportions of looking to target for these mispronunciations were at the same level as for correct pronunciations, i.e., equally successful recognition in both cases. On the other hand, recognition failed when T2 and T3 were mispronounced as T4; that is, toddlers detected the T2-to-T4 and T3-to-T4 mispronunciations. These results indicate that unlike T2-T3, the T2-T4 and T3-T4 contrasts are distinct at the lexical level for toddlers.

The failure to detect the T2-T3 contrast in Experiment 2 might be due to their acoustic similarity. The pitch patterns of T2 and T3 are the most similar among all tonal contrasts in Mandarin. Stager and Werker (1997) showed that during word learning, similar segments such as /b/-/d/ were not distinguished by 14-month-olds, although the effect was due to young word learners' temporary processing limitation under certain task conditions. The confusion was absent when the word-learning task was made easier or when slightly older infants (17-month-olds) were tested (e.g., Werker et al., 2002; Fennell and Werker, 2003; Yoshida et al., 2009). With regards to our Experiment 2, previous studies on familiar word comprehension are most pertinent for consideration. In a previous study on familiar word recognition in English by White and Morgan (2008), toddlers' looking to targets varied according to the degree of mispronunciation of the word onset consonant, with reduced mispronunciation effects for smaller phonetic deviances than larger ones, indicating graded lexical representations. It is possible that toddlers do not distinguish T2 and T3 in familiar words due to their acoustic/perceptual similarity, and have less sensitivity to this contrast during word comprehension.

However, toddlers' T2-T3 confusion differs from the broader evidence that phonetically similar consonants and vowels are distinguished in infants' earliest familiar words (e.g., Swingley and Aslin, 2002; Fennell and Werker, 2003; Mani and Plunkett, 2007). Notably, even the smallest deviances in the sensitive task of White and Morgan (2008) still yielded a mispronunciation effect (significantly less looking to target in 1-feature MP trials than in CP trials), meaning that the most similar contrasts remained discriminable for toddlers, the same as for adults. That is, continuity was maintained from phonetic discrimination in early infancy to subsequent phonological development and to mature representation in adults. Our toddlers, however, were very different. They showed a complete lack of any mispronunciation effect for T2-to-T3 and T3-to-T2 deviances. This result was at odds with the clear discrimination of T2 and T3 shown in Experiment 1. We note that T2-T3 stimuli were made more acoustically similar than usual in Experiment 1, but this did not impede discrimination. Moreover, T2-T3 and T2-T4 were discriminated equally in Tsao (2008), i.e., comparable in perceptual salience. However, our toddlers distinguished T2-T4 but totally confused T2-T3. This was striking since T2-T3 stimuli were more distinct acoustically in Experiment 2 than in Experiment 1. The results of our experiments suggest that there may be reasons beyond acoustically based perceptual salience for T2-T3 development, as discussed below.

The T2-T3 confusion may be because children had heard the same lexical items in both tones in the input due to Tone 3 Sandhi. Both tones occur in surface realizations for the same words, e.g., *xiao* in *xiao3tu4* “little bunny” vs. *xiao2ma3* “little horse” (the numbers here indicate the surface realizations of tones). In *xiao2ma3*, the underlying T3T3 sequence surfaces as T2T3 due to Tone 3 Sandhi. Children might store both variants of T3 (the low variant and the T2-like rising variant) for words such as *mai* (“buy”), *xiao* (“little”), *hao* (“good”). What is more complicated is that T3T3 does surface in certain syntactic structures, against Tone 3 Sandhi. For example, *bi3* and *ma3* remain as T3T3 when surfacing in [*gou2*-dog [[*bi3*-than *ma3*-horse] *kuai4*-fast]] “dogs are faster than horses” (Duanmu, 2007). Thus, by observing the tonal changes in some known words, children can overgeneralize T2 and T3 as free variations across words, neutralizing them as variants within one functional category. Our results are consistent with this possibility. The T2-T3 confusion has also been observed in older children, who did not detect T3-to-T2 and T2-to-T3 mispronunciations during word comprehension (Singh et al., 2017). Wong et al. (2005), however, showed in a different task that 3-year-olds advance in their understanding of Tone 3 Sandhi, thus confusing only the T3-to-T2 change but not vice versa in word recognition. This asymmetry resembles native Mandarin adult listeners' asymmetrical responses in the ERP study of Li and Chen (2015), consistent with Tone 3 Sandhi.

The two ideas, acoustic similarity and sandhi alternation, are in fact related. As described in the Introduction, neutralization rules in natural languages tend to occur for phonemes that are acoustically/phonetically similar, such as the cases of flapping in English, obstruent devoicing neutralization in German and Tone 3 Sandhi in Mandarin. The two ideas cannot be easily separated, and the results of Experiment 2 are consistent with both. Nevertheless, the results of Experiment 1 in the present study suggest that T2 and T3 are perceptually discriminable. Even non-tone-speaking teenagers can discriminate T2 and T3 as successfully as do Mandarin-speaking peers (Pierce et al., 2014), indicating that the contrast is sufficiently salient acoustically. Thus, the complete lack of any mispronunciation effect between T2 and T3 in our toddlers is likely due to phonological reasons such as neutralizations related to Tone 3 Sandhi.

In our word comprehension experiment we used only monosyllabic words to test T2 and T3. There are in fact many bisyllabic (and some trisyllabic) compound words containing T2 and T3 (e.g., *ping2guo3* “apple,” *yi3zi* “chair,” *tuo1xie2* “slipper,” *chang2jing3lu4* “giraffe”) that young toddlers know. Tone processing in compounds might be more challenging for learners due to coarticulation of neighboring tones that applies generally at the phonetic level (Gauthier et al., 2007a,b; Shi, 2009). It would be interesting to examine children's processing of lexical tones in compound words in future research.

In sum, our experiments show that during the first year of life tone-learning babies can discriminate similar lexical tones in their native language, as they do for similar consonant and vowel contrasts (e.g., Werker and Tees, 1984; Polka and Werker, 1994). However, during the second year of life when tones become organized in the developing lexicon, toddlers fail to distinguish similar tones in words, while they successfully represent dissimilar tonal contrasts in words. Toddlers' lexical representation seems to be affected by hearing words that go through neutralization in the input (also see recent work on consonant neutralization in Van der Feest and Johnson, 2016). A phonetic contrast that is acquired early in infancy seems to be reorganized and overgeneralized as one functional category (containing multiple variants) at the lexical stage, as toddlers focus on building a vocabulary and establishing a phonemic system.

## Ethics statement

This study was carried out in accordance with the recommendations of the Institute of Linguistics, CASS, China, with written informed consent from all subjects. All subjects gave written informed consent in accordance with the Declaration of Helsinki. The protocol was approved for ethics by the Institute of Linguistics, CASS, China.

## Author contributions

RS: Conceptualized and designed the study, supervised the construction and the execution of the experiments, directed the data analyses, and wrote the article. JG: Prepared/tested the experiments, coded/organized the data, and conducted the statistical analyses under the supervision of the first author. AA: Conducted further detailed statistical analysis of the data and participated in the writing. AL: Lead for obtaining the Chinese funding for the experimental procedure and the testing, and participated in discussions during the study.

### Conflict of interest statement

The authors declare that the research was conducted in the absence of any commercial or financial relationships that could be construed as a potential conflict of interest.

## Footnotes

^1^To verify if these fillers were indeed familiar words to our toddlers, we analyzed their comprehension of the named targets in filler trials within the same time window that was used for analyzing the key trials, i.e., 360–2,000 ms from the onset of the target word. For each filler word, the proportion of looking to the target was compared with the 0.5 chance level. We found that the toddlers indeed knew the filler words. Looking to targets was significantly above chance for all the filler words (*p* levels ranged from.000 to.022, two-tailed) except the one in the first trial of the whole experiment, which was expected since in the very first trial children were just getting acquainted with the equipment and the task.

## References

[B1] Anderson J. Morgan J. L. White K. S. (2003). A statistical basis for speech sound discrimination. Lang. Speech 46, 155–182. 10.1177/00238309030460020601 14748443

[B2] Ballem K. Plunkett K. (2005). Phonological specificity in children at 1;2. J. Child Lang. 32, 159–173. 10.1017/S0305000904006567 15779881

[B3] Chao Y. (1930). A system of tone letters. Le Maitre Phonetique 45, 24–27.

[B4] Csibra G. Hernik M. Mascaro O. Tatone D. Lengyel M. (2016). Statistical treatment of looking-time data. Dev. Psychol. 52, 521–536. 10.1037/dev0000083 26845505 PMC4817233

[B5] Duanmu S. (2007). Phonology of STANDARD CHINESE. Oxford: Oxford University Press.

[B6] Feldman N. H. Myers E. B. White K. S. Griffiths T. L. Morgan J. L. (2013). Word-level information influences phonetic learning in adults and infants. Cognition 127, 427–438. 10.1016/j.cognition.2013.02.007 23562941 PMC3646897

[B7] Fennell C. T. Werker J. F. (2003). Early word learners' ability to access phonetic detail in well-known words. Lang. Speech 46, 245–264. 10.1177/00238309030460020901 14748446

[B8] Gauthier B. Shi R. Xu Y. (2007a). Learning phonetic categories by tracking movements. Cognition, 103, 80–106. 10.1016/j.cognition.2006.03.002 16650399

[B9] Gauthier B. Shi R. Xu Y. (2007b). Simulating the acquisition of lexical tones from continuous dynamic input. J. Acoust. Soc. Am. 121, EL190–EL195. 10.1121/1.2716160 17550202

[B10] Hao M. Shu H. Xing A. Li P. (2008). Early vocabulary inventory for Mandarin Chinese. Behav. Res. Methods 40, 728–733. 10.3758/BRM.40.3.728 18697668

[B11] Harrison P. (2000). Acquiring the phonology of lexical tone in infancy. Lingua 110, 581–616. 10.1016/S0024-3841(00)00003-6

[B12] Hay J. F. Graf Estes K. Wang T. Saffran J. R. (2015). From flexibility to constraint: the contrastive use of lexical tone in early word learning. Child Dev. 86, 10–22. 10.1111/cdev.12269 25041105 PMC4295000

[B13] Kuhl P. K. Williams K. A. Lacerda F. Stevens K. N. Lindblom B. (1992). Linguistic experience alters phonetic perception in infants by 6 months of age. Science 255, 606–608. 10.1126/science.1736364 1736364

[B14] Li X. Chen Y. (2015). Representation and processing of lexical tone and tonal variants: evidence from the mismatch negativity. PLoS ONE 10:e0143097. 10.1371/journal.pone.0143097 26625000 PMC4666592

[B15] Mani N. Plunkett K. (2007). Phonological specificity of vowels and consonants in early lexical representations. J. Mem. Lang. 57, 252–272. 10.1016/j.jml.2007.03.005

[B16] Mattock K. Burnham D. (2006). Chinese and English infants' tone perception: evidence for perceptual reorganization. Infancy 10, 241–265. 10.1207/s15327078in1003_3

[B17] Maye J. Werker J. F. Gerken L. (2002). Infant sensitivity to distributional information can affect phonetic discrimination. Cognition 82, B101–B11. 10.1016/s0010-0277(01)00157-3 11747867

[B18] Pierce L. J. Kline D. Chen J. K. Delcencerie A. Genesee F. (2014). Mapping the unconscious maintenance of a lost first language. Proc. Natl. Acad. Sci. U.S.A. 111, 17314–17319. 10.1073/pnas.1409411111 25404336 PMC4260567

[B19] Polka L. Werker J. F. (1994). Developmental changes in perception of non-native vowel contrasts. J. Exp. Psychol. Hum. Percept. Perform. 20, 421–435. 10.1037/0096-1523.20.2.421 8189202

[B20] Shi R. (2009). Contextual variability and infants' perception of tonal categories. Chinese J. Phonet. 2, 1–9.

[B21] Shi R. Santos E. Gao J. Li A. (2017). Perception of similar and dissimilar lexical tones by non-tone-learning infants. Infancy. [Epub ahead of print]. 10.1111/infa.12191

[B22] Singh L. Foong J. (2012). Influences of lexical tone and pitch on word recognition in bilingual infants. Cognition 124, 128–142. 10.1016/j.cognition.2012.05.008 22682766 PMC3390932

[B23] Singh L. Goh H. H. Wewalaarachchi T. D. (2015). Spoken word recognition in early childhood: comparative effects of vowel, consonant and lexical tone variation. Cognition 142, 1–11. 10.1016/j.cognition.2015.05.010 26010558

[B24] Singh L. Hui T. J. Chan C. Golinkoff R. M. (2014). Influences of vowel and tone variation on emergent word knowledge: a cross-linguistic investigation. Dev. Sci. 17, 94–109. 10.1111/desc.12097 24118787

[B25] Singh L. Tan A. Wewalaarachchi T. D. (2017). Lexical tone variation and spoken word recognition in preschool children: effects of perceptual salience. J. Child Lang. 44, 924–942. 10.1017/S0305000916000325 27377868

[B26] So C. Best C. (2010). Cross-language perception of non-native tonal contrasts: effects of native phonological and phonetic influences. Lang. Speech 53, 273–293. 10.1177/0023830909357156 20583732 PMC2897724

[B27] Stager C. L. Werker J. F. (1997). Infants listen for more phonetic detail in speech perception than in word learning tasks. Nature 388, 381–382. 10.1038/41102 9237755

[B28] Swingley D. Aslin R. N. (2002). Lexical neighborhoods and the word-form representations of 14-month-olds. Psychol. Sci. 13, 480–484. 10.1111/1467-9280.00485 12219818

[B29] Tsao F.-M. (2008). The effect of acoustical similarity on lexical-tone perception of one-year-old Mandarin-learning infants. Chinese J. Psychol. 50, 111–124.

[B30] Van der Feest S. Johnson E. K. (2016). Input driven differences in toddlers' perception of a disappearing phonological contrast. Lang. Acquis. 23, 89–111. 10.1080/10489223.2015.1047096

[B31] Werker J. F. Fennell C. T. Corcoran K. Stager C. L. (2002). Infants' ability to learn phonetically similar words: effects of age and vocabulary size. Infancy 3, 1–30. 10.1207/S15327078IN0301_1

[B32] Werker J. F. Tees R. C. (1984). Cross-language speech perception: evidence for perceptual reorganization during the first year of life. Infant Behav. Dev. 7, 49–63. 10.1016/S0163-6383(84)80022-3

[B33] White K. S. Morgan J. L. (2008). Subsegmental detail in infants' early lexical representations. J. Mem. Lang. 59, 114–132. 10.1016/j.jml.2008.03.001

[B34] Wong P. Schwartz R. Jenkins J. (2005). Perception and production of lexical tones by 3-year-old, Mandarin-speaking children. J. Speech Lang. Hear. Res. 48, 1065–1079. 10.1044/1092-4388(2005/074) 16411796

[B35] Xu Y. (1997). Contextual tonal variations in Mandarin. J. Phonet. 25, 61–83. 10.1006/jpho.1996.0034

[B36] Xu Y. Wang Q. E. (2001). Pitch targets and their realization: evidence from Mandarin Chinese. Speech Commun. 33, 319–337. 10.1016/S0167-6393(00)00063-7

[B37] Yeung H. H. Chen K. H. Werker J. F. (2013). When does native language input reorganize phonetic perception? The precocious case of lexical tone. J. Mem. Lang. 68, 123–139. 10.1016/j.jml.2012.09.004

[B38] Yeung H. H. Werker J. F. (2009). Learning words' sounds before learning how words sound: 9-Month-olds use distinct objects as cues to categorize speech information. Cognition 113, 234–243. 10.1016/j.cognition.2009.08.010 19765698

[B39] Yip M. (2002). Tone. Cambridge: Cambridge University Press.

[B40] Yoshida K. A. Fennell C. T. Swingley D. Werker J. F. (2009). Fourteen month-old infants learn similar sounding words. Dev. Sci. 12, 412–418. 10.1111/j.1467-7687.2008.00789.x 19371365 PMC2883913

