# Supplementary material for: Perception and Representation of Lexical Tones in Native Mandarin-Learning Infants and Toddlers
Source: Front Psychol. 2017 Jul 21;8:1117. doi: 10.3389/fpsyg.2017.01117 (PMC5519614; doi:10.3389/fpsyg.2017.01117)

Appendix A

Stimuli of one of the four orders in Experiment 2

	Trial	Target word	Target image	Distractor	Distractor image
1	Filler	*mao1* ‘cat’	left	*gou3* ‘dog’	right
2	Filler	*qiu2* ‘ball’	left	*xie2* ‘shoe’	right
3	CP	*wan3* ‘bowl’	right	‘roller’	left
4	Filler	*ma3* ‘horse’	left	*yang2* ‘sheep’	right
5	Filler	*bao1* ‘bag’	left	‘roller’	right
6	Filler	*ji1* ‘chicken’	right	*yang2* ‘sheep’	left
7	Filler	*cai4* ‘vegetable’	right	*chuang2* ‘bed’	left
8	MP	*yang4* ‘sheep’	left	‘badger’	right
9	Filler	*chuang2* ‘bed’	right	*wan3* ‘bowl	left
10	Filler	*niu2* ‘cow’	left	*ma3* ‘horse’	right
11	CP	*yang2* ‘sheep’	right	‘badger’	left
12	Filler	*shu4* ‘tree’	left	*wan3* ‘bowl’	right
13	Filler	*shu1* ‘book’	left	*chuang2* ‘bed’	right
14	MP	*wan2* ‘bowl’	left	‘roller’	right
15	Filler	*deng1* ‘lamp’	right	*shu4* ‘tree’	left
16	Filler	*hua1* ‘flower’	right	*wan3* ‘bowl’	left
17	CP	*yang2* ‘sheep’	left	‘badger’	right
18	Filler	*chuang2* ‘bed’	left	*wan3* ‘bowl’	right
19	Filler	*zhu1* ‘pig’	right	*ma3 ‘horse’*	left
20	MP	*wan4* ‘bowl’	right	‘roller’	left
21	Filler	*yu2* ‘fish’	left	*yang2* ‘sheep’	right
22	CP	*wan3* ‘bowl’	left	‘roller’	right
23	Filler	*cai4* ‘vegetable’	left	*qiu2* ‘ball’	right
24	MP	*yang3* ‘sheep’	right	‘badger’	left
25	Filler	*xie2 ‘shoe’*	right	*chuang1 ‘window’*	left
26	Filler	*shu4 ‘tree’*	left	‘badger’	right
27	Filler	*ma3 ‘horse’*	right	*yang2* ‘sheep’	left
28	Filler	*hou2 ‘monkey’*	right	*tu4 ‘rabbit’*	left

Appendix B

Time-normalized F0 trajectories of T2-*yang2* and T3-*wan3* correct pronunciations and mispronunciations (T2 mispronounced as T3 and T4, and T3 mispronounced as T2 and T4) in Experiment 2

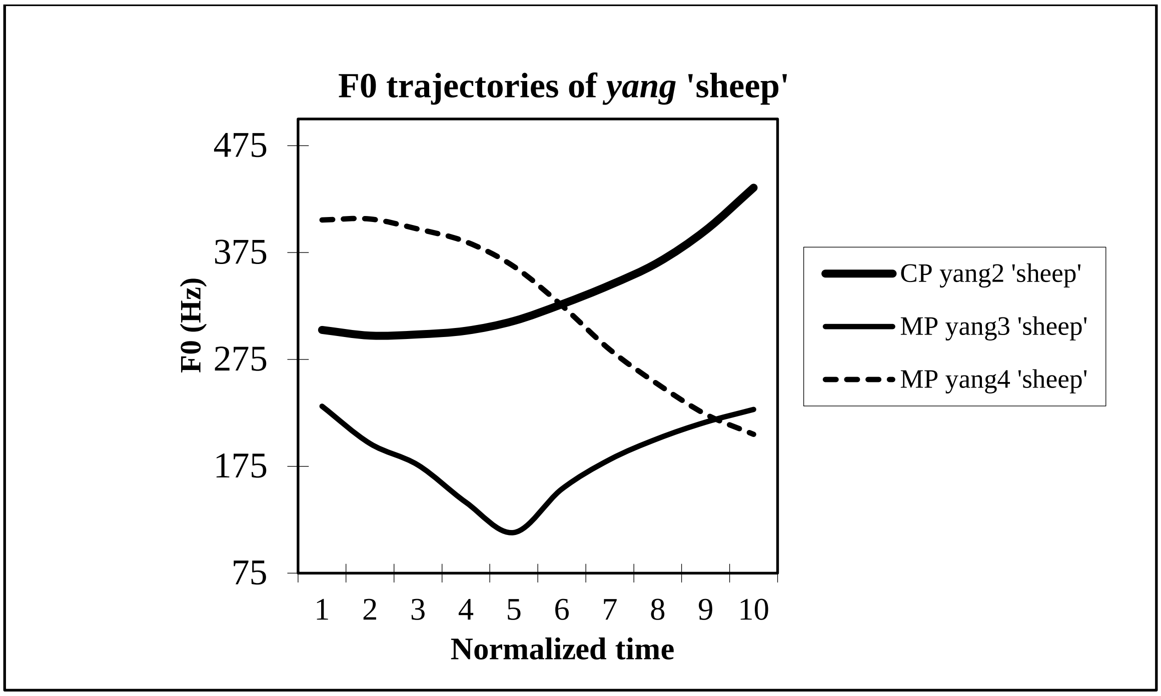

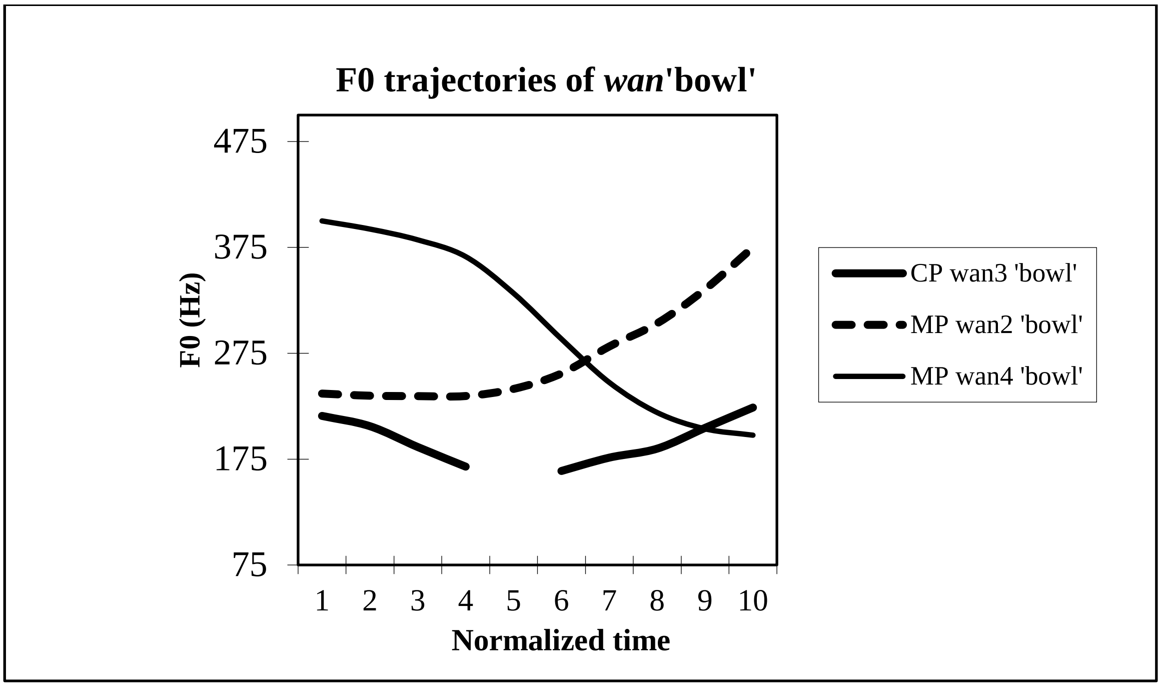

Supplement: Supplementary file 1 [file DataSheet1.pdf]
